# A survey on the knowledge and attitudes of pharmacists towards the application of antimicrobial therapeutic drug monitoring and its challenges in Qatar

**DOI:** 10.1371/journal.pone.0297699

**Published:** 2024-02-27

**Authors:** Dania Ihsan Alkhiyami, Alya Salah Higazy, Mohamed Omar Saad

**Affiliations:** Clinical Pharmacy Department, Al-Wakra Hospital, Hamad Medical Corporation, Doha, Qatar; Garraham Pediatric Hospital (Hospital de Pediatria Prof. Dr. Juan Garraham), ARGENTINA

## Abstract

**Introduction:**

Therapeutic drug monitoring (TDM) is an integral part of pharmaceutical care. Antimicrobials are amongst the most commonly monitored medications. Therefore, identifying the gaps in antimicrobial pharmacokinetics and TDM knowledge and skills among pharmacists is crucial to optimize TDM application.

**Research question:**

What is the current knowledge, attitudes and perceived barriers of pharmacists in Qatar towards the application of antimicrobial TDM?

**Study design:**

Cross-sectional survey.

**Methods:**

The psychometric validation of the survey underwent 3 stages: domain identification and item generation, content validation, and pilot test. The survey was divided into 4 domains (participant characteristics, knowledge, attitudes, and perceived barriers). It was developed in Survey Monkey and distributed to all pharmacists in Hamad Medical Corporation (HMC) hospitals via email. Data was analyzed using IBM Statistical Package for the Social Sciences (SPSS). Categorical and quantitative variables were expressed as frequencies with percentages and medians with interquartile ranges, respectively. Mann–Whitney U-test was used to test the effect of demographic and professional parameters on the knowledge scores. P values less than 0.05 were considered significant.

**Results:**

Forty-nine responses were collected. The median age of respondents was 34 years and 51% of them were males. Most respondents were clinical pharmacists (47%). On average, 44% of knowledge questions were correct, whereas 32% were incorrect and 23% were not sure of the answer. The median knowledge score was 5 out of 10 (interquartile range 2.5–6). Participants with post-graduate degrees or prior pharmacokinetic training showed trends towards higher knowledge scores. Online pharmacokinetics calculators were the most frequently used dose adjustment method. The top perceived barriers for the implementation of antimicrobial TDM were lack of knowledge and lack of educational sessions.

**Conclusions:**

Albeit pharmacists in Qatar had modest level of knowledge about antimicrobial TDM, they had positive attitudes towards TDM and its implications in the clinical practice. Future plans should include providing TDM-related education activities.

## Introduction

Therapeutic drug monitoring (TDM) is the measurement of drug concentrations at particular times to maintain therapeutic concentrations in the blood and individualize dosing regimens [1, 2]. The knowledge and skills of clinical pharmacists in pharmacokinetics (PK) and pharmacodynamics of drugs put them in a better position to provide clinical services in the area of antimicrobial TDM. Over the last decades, the clinical pharmacist’s role has evolved and become the standard of care in different disciplines, such as pharmacokinetics, anticoagulation, antimicrobial stewardship, and medication therapy management [3–8]. Pharmacist-led TDM services have demonstrated a positive impact on patient care; it was found to reduce the incidence of drug side effects, reduce the length of therapy and hospital stay, and reduce morbidity and mortality, in addition to cost-savings [9–13]. However, the application of antimicrobial TDM is not without challenges. A major barrier to the application of antimicrobial TDM is healthcare practitioners’ knowledge and experience within this area. In Qatar, published studies assessing hospital pharmacists and TDM services are limited. A survey was conducted to evaluate the training backgrounds and attitudes of pharmacists in Qatar toward applying PK and revealed that formal education about TDM during college is generally perceived as lacking [14]. Moreover, the identified barriers included spending time on inventory and dispensing rather than patient-care services such as TDM and lack of practical pharmacokinetic knowledge [14]. Antimicrobials have been the most commonly monitored medications, and significant research has been performed on the antimicrobial’s concentration–effect relationships [15, 16]. Optimizing antimicrobial dosing is essential to improve clinical outcomes, reduce resistance, and minimize toxicity [17]. Therefore, identifying pharmacist’s limitations in antimicrobials pharmacokinetics and TDM knowledge and skills will help plan and implement antimicrobial TDM courses that address these limitations and, consequently, improve patient outcomes. The current study aimed to assess the knowledge and attitudes of pharmacists toward the application of antimicrobial therapeutic drug monitoring services and its challenges in Qatar. This study was approved by Hamad Medical Corporation (HMC) Medical Research Center (MRC-01-19-145).

## Materials and methods

This study was a cross-sectional, descriptive study that was conducted using a web-based survey. The recruitment period was from 27/06/2019 to 11/12/2019. The study targeted licensed hospital pharmacists practicing at HMC hospitals. HMC is a governmental health system in Qatar and is considered the leading provider of secondary and tertiary health care services in Qatar. The survey was designed to be applicable and convenient for HMC clinical settings. This survey aimed to assess the knowledge and attitude of Qatar pharmacists working in HMC toward antimicrobial TDM and identify the perceived barriers. The validity of the survey was tested by following the stages of psychometric validation: (1) Domain identification and item generation: Three investigators developed the items after doing a thorough literature review on applying TDM services and the perceptions of pharmacists on their role. Then, the first panel took place to refine and group items into domains. The items were reviewed and divided into four main domains (participant characteristics, level of knowledge, attitudes, and perceived barriers). The first domain was refined to contain ten items. In this domain, we aimed to collect the professional characteristics in terms of the participants’ academic degrees, positions, facilities, and whether they received any previous TDM-related certification. The second domain consisted of ten items assessing general knowledge of the antimicrobial TDM. A score of one point was assigned if the correct answer was chosen, and a score of zero points if the wrong answer or ’Not sure’ was chosen. The third domain was designed to evaluate the attitudes of participants towards antimicrobial TDM and its application. It consisted of two parts with eight and five items, respectively. The responses of the first part were on a 5-point Likert scale ranging from strongly agree to disagree strongly. The responses of the second part were on a 5-point scale ranging from always to never, with an additional option if the respondent is unsure about the dosing method. The last domain in the survey evaluated the perceived barriers to implementing antimicrobial TDM in HMC. A list of barriers was provided, and participants were allowed to choose one or more, as well as an option to write down any other perceived barriers. (2) Content validation: The second panel took place, and four expert members were contacted via email and invited to review and assess the clarity of the items’ construction and wording to ensure the comprehensiveness and content validity of the survey. The experts were asked to indicate the extent to which they perceive each item to represent the domain and readability and clarity of the questionnaire. Two expert members were pharmacy professors from Qatar University with long experience in pharmacokinetics; one was a clinical pharmacokinetics professor and research expert from Chicago, and one was a national pharmacokinetics research expert. The survey was modified in terms of the content and structure according to the feedback of the members of this panel. (3) Pilot test: This was done to measure the extent to which the items and scale of measurements could provide data to satisfy the study’s objectives and assess reliability. The generated survey was administered to a sample of 5 pharmacists from the targeted population (HMC pharmacists). Participants were instructed to complete questionnaires independently, and then the assigned investigator interviewed the participants and took their written and verbal feedback. There were no significant issues related to the survey and scales used. According to the comments of one of the pharmacists, the second question of the third domain (attitude) was to change the placement of "not sure about this method to next to never, not next to always." Otherwise, the feedback was that the questions were straightforward. It took participants approximately 12 minutes to complete the survey. Along with the survey, a research information sheet was attached, explaining the purpose and objectives and inviting the pharmacists to participate. It also ensured that participation is anonymous. The survey was distributed to and collected from all eligible participants practicing in HMC hospitals. An email was sent with a link to the survey developed in Survey Monkey, and the responses were collected through the link; their response to the survey was considered consent from the participants.

## Statistical analysis

Data were exported from Survey Monkey and analyzed using IBM *Statistical Package for the Social Sciences* (SPSS) for Windows, version 23 (IBM et al., N.Y., USA). Categorical variables were expressed as frequencies and percentages, including respondents’ demographics and professional characteristics, and items assessing knowledge and attitudes, and perceived barriers. Median and interquartile ranges were used to summarize quantitative variables, including knowledge scores. Mann–Whitney U-test was used to test the effect of demographic and professional parameters on the knowledge scores. Pictorial presentations of key results were made using appropriate statistical graphs. All P values presented were two-tailed, and P values less than 0.05 were considered statistically significant.

## Results and discussion

### Pharmacists characteristics

Total of 450 pharmacists were contacted through an electronic survey link sent to all pharmacists working at HMC via email. Over a period of six months, 49 responses were collected (∼11% overall response rate). Of these 49 respondents, 25 (51%) were males and 24 (49%) were females. Most of the respondents were clinical pharmacists (47%). Median age of participants was 34 years, with a median of 7 years of practice in HMC. Most respondents (67.3%) indicated they have extra credentials besides a bachelor’s degree. Around 10% of the respondents completed clinical pharmacokinetics/TDM-related certification. Table 1 summarizes the participants’ demographics, characteristics, and professional information.

**Table 1:** Demographic and practice characteristics.

Characteristic	N = 49
Age (year)–median (IQR)	34 (30–38)
Number of years in HMC (year)–median (IQR)	7 (1–11)
Male gender–no. (%)	25 (51)
Area of practice (N = 49)	Frequency (Percent)
Pharmacy Operation	13 (27%)
General Medicine	12 (25%)
Emergency Medicine/critical care	5 (10%)
Cardiology	4 (8%)
General Pediatrics	3 (6%)
Infectious Disease	3 (6%)
Obstetrics/Gynecology	2 (4%)
Oncology/Hematology	1 (2%)
Others	6 (12%)
Post-graduate degree—no. (%)	33 (67.3)
Doctor of Pharmacy	16 (32.7)
Master’s degree	16 (32.7)
Doctor of Philosophy	1 (2)
Clinical pharmacokinetics/therapeutic drug monitoring related certification- no. (%)	4 (8)

### Knowledge about TDM

**Fig 1:**
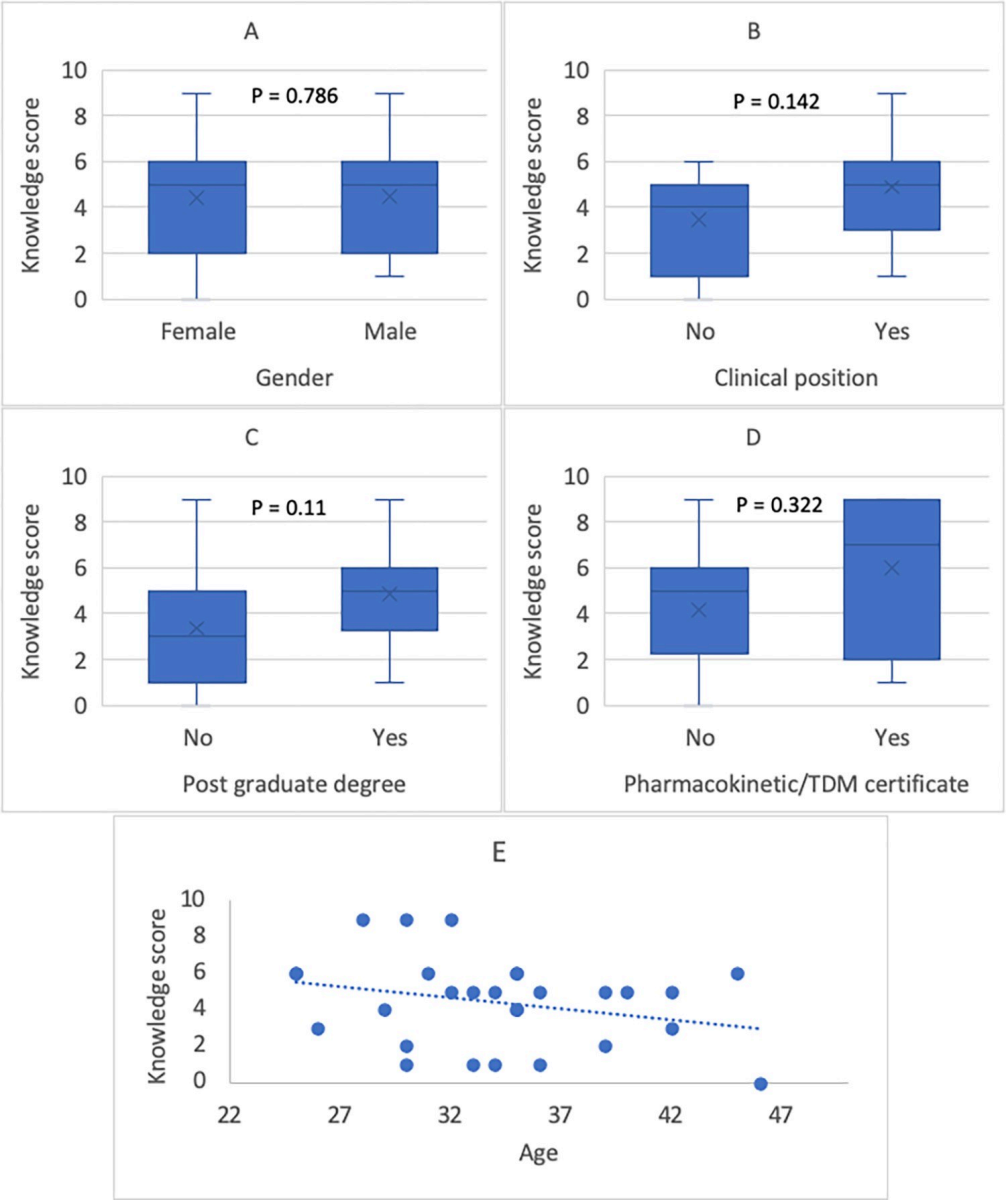
The influence of the different characteristics and profession on the scores of knowledges about TDM. A: The effect of gender on the knowledge score; B: The effect of clinical position on the knowledge score; C: The effect of holding a post graduate degree on the knowledge score; D: The effect of pharmacokinetic/TDM certification on the knowledge score. E. The effect of age on the knowledge score.

Fifty-seven percent of respondents completed questions pertaining to knowledge about TDM. On average, 44% of completed answers were correct, 32% were incorrect, and 23% needed clarification. We have explored the factors that may influence pharmacist knowledge of TDM (Fig 1A–1D).

Non-statistically significant higher knowledge scores were observed in pharmacists with a postgraduate degree (Doctor of Pharmacy (PharmD) or master’s degree) compared to a bachelor’s degree (median score five vs. three). PharmD degree holders had slightly higher knowledge scores when compared to master’s degree holders (median score 5.5 vs. 5). Prior pharmacokinetic training had an apparent positive impact on knowledge scores (median score seven vs. five). In addition, younger age was associated with a trend toward a higher knowledge score (Fig 1E).

### Attitudes towards TDM and its implications in practice

Table 2 illustrates pharmacists’ attitudes towards TDM and its implications in Qatar clinical practice. Eight items on attitudes are presented on a 5-point Likert scale: ’Strongly agree; agree; neutral; disagree; strongly disagree. Pharmacists had positive attitudes towards TDM and its implications as most respondents agreed or strongly agreed with all eight attitude statements. Most of the respondents (88%) agreed or strongly agreed that it is their responsibility to apply TDM for recommending the optimum dose of antimicrobials. More than half of the respondents agreed or strongly agreed that they encounter difficulty in applying TDM knowledge for antimicrobials in practice and that there is a need for TDM consultation service in the hospital for complicated cases (60% and 70%, respectively). Moreover, around 80% of the respondents expressed interest in undertaking antimicrobial TDM educational sessions.

**Table 2:** Attitudes towards TDM and its implications in the clinical practice.

Questions	Frequency (percent)				
	What is your extent of agreement with the following statements?				
	Strongly agree	Agree	Neutral	Disagree	Strongly Disagree
It is important for me as a pharmacist to identify antimicrobials that require therapeutic drug monitoring (TDM)	19 (76%)	4 (16%)	1 (4%)	0 (0%)	1 (4%)
It is my professional responsibility to apply TDM for recommending the optimum dose of antimicrobials	18 (72%)	4 (16%)	2 (8%)	0 (0%)	1 (4%)
I believe that TDM of antimicrobial may improve patient clinical response	18 (72%)	4 (16%)	2 (8%)	1 (4%)	0 (0%)
I believe that TDM of antimicrobial may minimize the risk of dose-related side effects	18 (72%)	5 (20%)	2 (8%)	0 (0%)	0 (0%)
I believe that TDM of antimicrobial will improve our ability to effectively control drug therapy expenditures	13 (54%)	7 (29%)	3 (13%)	1 (4%)	0 (0%)
I encounter difficulty in applying TDM knowledge for antimicrobials in practice	7 (28%)	8 (32%)	5 (20%)	5 (20%)	0 (0%)
I believe there is a need for TDM consultation service in the hospital for complicated cases	15 (60%)	4 (16%)	3 (12%)	3 (12%)	0 (0%)
I am interested as a health care professional to undertake antimicrobial TDM training sessions/ workshops	16 (64%)	4 (16%)	1 (4%)	4 (16%)	0 (0%)

Table 3 shows pharmacists’ utilization and awareness of different dose adjustment methods in clinical practice.

**Table 3:** Dose adjustment methods in current practice.

Questions	Frequency (percent)				
	On average, how often do you use the dose adjustment methods after TDM in your practice for decision making:				
	Always	Often	Rarely	Never	Not aware about this method
Use online PK calculators	6 (24%)	7 (28%)	7 (28%)	4 (16%)	1 (4%)
Empirically adjust antimicrobial dose without calculations	4 (16%)	7 (28%)	10 (40%)	4 (16%)	0 (0%)
Use model-specific PK calculations.	4 (16%)	5 (21%)	8 (34%)	5 (21%)	2 (8%)
Use Bayesian dose optimizing software	4 (16%)	2 (8%)	3 (12%)	11 (44%)	5 (20%)
Use linear PK to adjust doses (cross multiplication)	2 (8%)	10 (40%)	9 (36%)	3 (12%)	1 (4%)

The respondents indicated online PK calculators are the most used dose adjustment methods in clinical practice. Over half of the respondents were unaware of or had never used Bayesian dose optimization software. More than 30% of the respondents always or often empirically adjust antimicrobial doses without calculations. With regards to current TDM practice, more than half of the respondents (56%) rated the quality of current practice as 3 out of 5, while none rated the quality as 5 (highest rate indicating optimum service).

### Perceived barriers on the implementation of antimicrobial TDM in HMC

**Fig 2:**
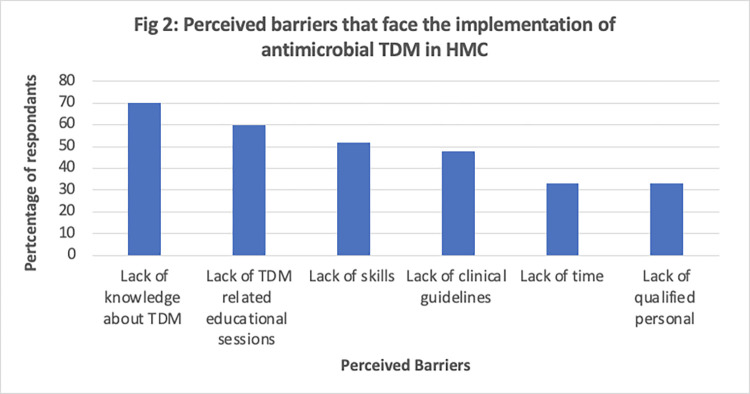
The perceived barriers that face the implementation of antimicrobial TDM in HMC, Qatar.

Most respondents indicated that the lack of knowledge about TDM (70%) and lack of TDM-related educational sessions (60%) are barriers to implementing antimicrobial TDM in HMC. Moreover, 55% of respondents indicated that the lack of skills (52%) was a barrier. Other frequently identified barriers were lack of clinical guidelines, time, and a need for qualified personnel. Fig 2 summarizes the perceived barriers to implementing antimicrobial TDM in HMC.

This study marks the inaugural effort in Qatar to evaluate the knowledge, attitudes, and barriers of pharmacists concerning antimicrobial TDM. The study targeted all actively practicing pharmacists across 12 HMC facilities which is Qatar’s main secondary and tertiary healthcare provider. The American Society of Hospital Pharmacy has endorsed TDM as an integral part of pharmaceutical care, whenever indicated [14]. The findings from this study help identify the deficiencies within pharmacists’ TDM practices in Qatar.

Within this study, pharmacists expressed a recognition of their role in recognizing antimicrobials requiring TDM. However, the overall knowledge of antimicrobial TDM among the participating professionals was found to be modest. Alongside self-reporting, the study assessed antimicrobial TDM knowledge using a set of questions that tackled fundamental and applicable concepts in pharmacokinetics. These questions were created by a panel of experienced pharmacists with a background in clinical pharmacokinetics practice and/or instruction. Over half of the provided responses in TDM knowledge domain were erroneous or indicated uncertainty, which is similar to the findings by Kheir et al.. In their study, they reported that 43% of respondents did not complete the knowledge section of the survey. Clinical pharmacists and pharmacists with prior pharmacokinetic certifications were more likely to answer, indicating a potential lack of confidence in TDM understanding.

Likewise, earlier studies have showed that lack of knowledge and training constituted a barrier to the provision of pharmacokinetics services [14]. Kheir et al. highlighted that 60% of hospital pharmacists identified lack of practical knowledge as a barrier to implementing pharmacokinetic principles, while 40% noted an poor understanding of pharmacokinetics as another obstacle. In a study exploring pharmacists’ perceptions of prescription drug monitoring programs, the authors concluded that training programs improve knowledge and motivate behavioral change [18]. Thus, educational initiatives concentrated on therapeutic drug monitoring are highly warranted.

The low response rate limited our ability to determine the factors affecting knowledge scores among our study cohort. However, a trend towards higher knowledge scores among pharmacists with postgraduate qualifications, clinical pharmacists, and/or pharmacists with prior pharmacokinetic certifications. Additionally, an inverse relationship of knowledge scores and age was observed. This could be due to the recent integration of evidence-based medicine and practical clinical pharmacokinetics in pharmacy curricula, along with the advancement of clinical pharmacists’ roles [19]. These trends underscore the significance of TDM education, which is supported by higher knowledge scores association with prior pharmacokinetic certifications. Furthermore, Moore and Tonnies have illustrated the potential for pharmacists with baccalaureate-level degrees to provide specific pharmacokinetic dosing services upon targeted instruction [20].

Over half of the respondents assigned a middling rating of 3 out of 5 to the current TDM practices at HMC, while none awarded the highest rating of 5. This indicates a need for enhancing TDM services at the institution. When evaluating pharmacists’ familiarity with diverse dose adjustment approaches, it was noted that 44% of pharmacists frequently adjusted antimicrobial doses without resorting to calculations, a practice diverging from the ASHP’s statement on the pharmacist’s role in clinical pharmacokinetic monitoring. Moreover, 64% of pharmacists were not aware of or had never utilized the Bayesian dose adjustment method. This could be attributed to limited access to Bayesian dose adjustment software and a clear demand for continuous educational sessions on this subject. Notably, a recent consensus guideline on vancomycin TDM for managing methicillin-resistant Staphylococcus aureus infections advocated for Bayesian software programs as the favored means for monitoring vancomycin’s area under the curve (AUC) [21]. Consequently, bridging this knowledge gap becomes an urgent need.

Pharmacists view prescribing the optimal antimicrobial doses to be integral part of their professional role, and they exhibit positive attitudes for engaging in training sessions and workshops focused on antimicrobial TDM. This aligns with the ASHP’s statement emphasizing TDM as an integral aspect of pharmaceutical care [1]. Furthermore, pharmacists have positive perceptions of the efficacy of antimicrobial TDM in enhancing their ability to effectively manage drug therapy costs and mitigate dose-related adverse effects. Earlier studies have provided evidence of the advantages of pharmacist-led TDM services, including a reduction in drug adverse effects, therapy duration, hospital stays, and overall morbidity and mortality rates, in addition to cost savings [9–13].

Perceived barriers to the implementation of antimicrobial TDM at HMC predominantly included lack of TDM knowledge and limited TDM-related educational sessions. A comparable pattern was observed in a prior survey that assessed the barriers to pharmacists’ application of pharmacokinetic knowledge in clinical contexts. Other challenges, acknowledged by approximately half of the respondents, included inadequate skills and a scarcity of clinical guidelines. The absence of a standardized protocol or guideline contributes to variability in antimicrobial prescribing and monitoring, thereby increasing the potential for errors in dosing, sampling times, and dose adjustments [22].

It is important to acknowledge several limitations of this study, with the foremost being the small sample size. Despite multiple reminders, the response rate remained low at 11%, potentially introducing bias. Moreover, nearly half of the surveyed pharmacists left several questions unanswered, limiting the generalizability of findings to all hospital pharmacists in Qatar and undermining the formulation of definitive conclusions. Additionally, the validity of the assessment questions employed to assess pharmacists’ knowledge of antimicrobial TDM remains to be established by future research.

## Conclusions

While the knowledge level of pharmacists concerning antimicrobial TDM was modest, they displayed favorable attitudes towards TDM and its relevance in clinical practice within Qatar. The primary obstacles perceived for integrating antimicrobial TDM at HMC were the lack of TDM knowledge and the scarcity of TDM-focused educational sessions. These results should serve as a compass for shaping the advancement of pharmacokinetic services and educational initiatives aimed at catering to pharmacists’ needs regarding antimicrobial TDM.

## Supporting information

S1 Data (XLSX)
