# Correction: A survey on the knowledge and attitudes of pharmacists towards the application of antimicrobial therapeutic drug monitoring and its challenges in Qatar

**DOI:** 10.1371/journal.pone.0313196

**Published:** 2024-10-31

**Authors:** Dania Ihsan Alkhiyami, Alya Salah Higazy, Mohamed Omar Saad

The published funding statement is incorrect. The correct funding statement is as follows: The authors would like to thank Hamad Medical Research Center (MRC) for covering the article processing charges (APC) for this publication and funding this study.

## References

[1] Alkhiyami DI, Higazy AS, Saad MO (2024) A survey on the knowledge and attitudes of pharmacists towards the application of antimicrobial therapeutic drug monitoring and its challenges in Qatar. PLoS ONE 19(2): e0297699. doi: 10.1371/journal.pone.0297699 38412165 PMC10898731

